# Protein Corona Stability and Removal from PET Microplastics: Analytical and Spectroscopic Evaluation in Simulated Intestinal Conditions

**DOI:** 10.3390/foods14203454

**Published:** 2025-10-10

**Authors:** Tamara Lujic, Tamara Mutic, Ana Simovic, Tamara Vasovic, Stefan Ivanovic, Maja Krstic Ristivojevic, Vesna Jovanovic, Tanja Cirkovic Velickovic

**Affiliations:** 1 Center of Research Excellence in Molecular Food Sciences and Department of Biochemistry, University of Belgrade—Faculty of Chemistry, Studentski trg 12-16, 11000 Belgrade, Serbiatmutic@chem.bg.ac.rs (T.M.); tvasovic@chem.bg.ac.rs (T.V.); krstic_maja@chem.bg.ac.rs (M.K.R.); vjovanovic@chem.bg.ac.rs (V.J.); 2 Institute of Chemistry, Technology and Metallurgy, National Institute of the Republic of Serbia, Department of Chemistry, University of Belgrade, Njegoseva 12, 11000 Belgrade, Serbia; stefan.ivanovic@ihtm.bg.ac.rs; 3 Serbian Academy of Sciences and Arts, Knez Mihailova 35, 11000 Belgrade, Serbia

**Keywords:** protein hard corona, microplastics, polyethylene terephthalate, polymer integrity, ATR FTIR spectroscopy

## Abstract

Microplastics entering the gastrointestinal environment rapidly acquire protein coronas that alter their surface chemistry and analytical detectability. We investigated the physicochemical interactions between fluorescently labeled bovine serum albumin (BSA) and polyethylene terephthalate (PET) microplastics during simulated intestinal exposure and evaluated the stability of the resulting hard corona. Using fluorescence tracking, SDS-PAGE, and FTIR spectroscopy, we showed that BSA forms a persistent corona that resists oxidative-only treatments. Only a combination of oxidation with an alkaline (KOH) or surfactant step (SDS) effectively removed the corona. None of the protocols applied affected polymer integrity. Residual protein in less effective protocols did not show changes on PET spectra in ATR FTIR. To validate the protocol under physiologically relevant complexity, we extended it to PET incubated with single digestive enzymes. FTIR spectra confirmed the removal of protein-specific signals in both systems, with no degradation of PET ester or aromatic functional groups nor signals of protein–polymer interactions. Our results highlight the robustness of protein–PET interactions in biological conditions and provide a variety of protocols for protein corona removal, suitable for diverse applications of microplastic analysis and toxicological studies.

## 1. Introduction

Plastic food packaging materials readily release microplastics (MPs) from packaging into foods, contributing to human exposure to MPs [1]. MPs are of particular concern due to their long-term durability in the environment and great potential for releasing plastic oligomers, additives, and chemicals and their vector capacity for adsorbing or collecting other pollutants [2]. Once introduced into aqueous or biological environments, MPs readily acquire surface-bound biomolecular layers, so-called “protein coronas” comprising loosely bound (soft) and tightly adsorbed (hard) proteins. These coronas may alter particle size, surface chemistry, and biological reactivity, complicating downstream analysis and influencing toxicological outcomes [3]. A mechanistic understanding of protein–MP interactions is therefore essential for assessing MP behavior in complex biological matrices and for developing effective protocols to isolate or characterize MPs in digested food, serum, or tissue samples.

Recent studies have begun to explore how MPs behave in simulated digestive environments, with a particular focus on the intestinal phase. For instance, Stock et al. [3] demonstrated that MPs incubated in simulated intestinal fluids formed stable bio-coronas composed of bile salts and digestive enzymes, significantly altering their colloidal stability and cellular uptake. Similarly, Schöpfer et al. [4] found that protein adsorption onto MPs persisted during digestion, supporting the presence of resilient hard coronas even under harsh enzymatic conditions and influencing subsequent analytical outcomes, including particle detection and interaction profiling. In biological matrices, such as digested food or serum, MP coronas can interfere with size characterization, compositional analysis, and surface reactivity assessments [3,4]. Such protein coatings can persist even after exposure to digestive enzymes, forming so-called “hard coronas” that resist removal and may bias analytical detection and toxicological interpretation. These findings reinforce the importance of understanding corona stability under gastrointestinal conditions, especially when designing effective decontamination protocols for analytical or toxicological studies.

Among the various types of MPs, polyethylene terephthalate (PET) is frequently found in food packaging, bottled water, and environmental samples [5]. Its relatively hydrophobic and chemically stable surface makes it prone to interacting with proteins such as bovine serum albumin (BSA), especially under physiologically relevant conditions like digestion. BSA is widely used as a model protein in studies of MP corona formation due to its well-characterized structure, amphiphilic properties, and relevance in mimicking biological fluid composition [6]. BSA shares structural and surface interaction characteristics with human serum albumin and digestive enzymes, making it a representative surrogate for protein adsorption studies [7]. Its ability to form stable hard coronas on hydrophobic surfaces allows for a controlled evaluation of adsorption mechanisms and removal strategies under physiologically relevant conditions. Furthermore, BSA’s widespread availability and stability under varied pHs and temperatures make it ideal for an in vitro simulation of gastrointestinal exposure [7]. The PET backbone is composed of terephthalic acid and ethylene glycol moieties linked via an ester bond, making it susceptible to degradation by hydrolytic enzymes that have been discovered in both bacteria and eukaryotes. Conversely, no functional enzymes have been found to degrade other plastic materials containing a hydrocarbon backbone, such as polypropylene and polystyrene [8]. Furthermore, these enzymes share a common mechanism of action, a catalytic triad consisting of serine, histidine, and a negatively charged amino acid [9], which is also found in pancreatic lipase and serine proteases [10], making PET an interesting candidate for studying the effects of gastrointestinal digestion.

The exposure of PET MPs to simulated digestive fluids can lead to measurable, although generally minor, spectral changes detectable by FTIR and Raman spectroscopy. These changes may include shifts or broadening in characteristic PET bands, such as the ester carbonyl stretch (~1715–1725 cm^−1^) and benzene ring modes (~1408–1450 cm^−1^), attributed to plastic–protein or plastic–lipid interactions in the intestinal environment [3,4]. Raman spectroscopy can also reveal reduced peak intensity or subtle shifts due to surface adsorption or mild oxidative changes [11]. Nevertheless, the core polymeric structure of PET generally remains intact, suggesting that gastrointestinal digestion does not induce significant degradation.

Recent advances in the clean-up of protein coronas on microplastics under simulated gastrointestinal conditions have increasingly focused on combining chemical and biological strategies. Proteolytic enzymes such as trypsin and proteinase K have been reported to selectively degrade adsorbed proteins [12], while oxidative approaches including hydrogen peroxide, Fenton reactions, and photocatalysis improve the removal of more persistent corona layers [13]. Protocols based on surfactants remain widely used, but their efficiency can be enhanced when applied together with enzymatic or alkaline treatments, thus minimizing residual protein coverage while preserving polymer integrity [14,15]. Collectively, these developments highlight the need for tailored protocols that address the complexity of corona formation in simulated intestinal fluid and ensure the reliable downstream spectroscopic identification of polymers. In this study, we use fluorescently labeled BSA to quantify removal efficiency after corona formation on PET MPs in simulated intestinal fluid conditions. By comparing three clean-up protocols—ionic detergent and hydrogen peroxide, hydrogen peroxide oxidation, and hydrogen peroxide combined with an alkali—we assessed both removal efficiency and the mechanisms of protein–polymer interaction in conditions simulating intestinal fluid. Quantification by fluorescence, protein analysis by SDS-PAGE, and structural confirmation via FTIR provide an integrated view of corona stability and clean-up effectiveness. Furthermore, we confirmed that layers of hard bound protein corona do not interfere with downstream characterization nor affect polymer identification and characterization. Additionally, relevant conditions were applied to investigate the removal efficiency of trypsin, chymotrypsin, and lipase coronas, as well as the effect of these digestive enzymes on the spectral changes in PET.

## 2. Materials and Methods

### 2.1. Materials

Polyethylene terephthalate (PET) microplastics (MPs) (<80 µm) were prepared at the University of Vienna. Powders of food-grade PET (CAS:25038-59-9) were produced using a ZM 200 ultra-centrifugal mill (Retsch GmbH, Haan, Germany) with an 80 μm ring sieve with trapezoid holes (03.647.0465) and cyclone accessories. MP fractions <80 µm were collected and kept at 4 °C as a dry powder. The characteristics of the obtained PET MPs were described in detail by Lujic et al. [16].

Potassium hydroxide (KOH), sodium dodecyl sulfate (SDS), hydrogen peroxide (H_2_O_2_, 30%), and absolute ethanol (EtOH, HPLC grade, Merck, Darmstadt, Germany) were of analytical grade and used in three clean-up protocols. Bovine serum albumin (BSA) (cat. no. A7906), lipase from porcine pancreas (cat. no. L3126-100G), and chymotrypsin (cat. no. C4129) were obtained from Sigma Aldrich (St. Louis, MI, USA) and used for the preparation of the PET MP protein’s corona. Before preparing the MP-BSA corona, BSA was conjugated with AlexaFluor488 (AF488) fluorescent dye (BSA-AF488) using an AF488 protein labeling kit (Invitrogen, Molecular Probes, Inc., Eugene, OR, USA) according to the manufacturer’s instructions. Briefly, 75 µL amino-reactive dye AF488 solution in dimethyl sulfoxide (10 mg/mL) was slowly added in 1 mL BSA solution (conc. 10 mg/mL in 0.1 M sodium bicarbonate buffer pH 8.3). After 1 h incubation at room temperature (RT), BSA-AF488 was separated from unbound AF488 by gel filtration using Sephadex G-25 (Sigma Aldrich, St. Louis, MI, USA). After chromatography, BSA-AF488 protein concentration was determined using Bicinchoninic acid (BCA) Protein Assay Kit (Pierce Biotechnology, Rockford, IL, USA). Enzyme solutions were freshly prepared following the procedures described below.

A PVDF membrane filter, 0.22 μm, (Millipore, Merck, Darmstadt, Germany) and stainless steel filter with a pore size of 10 μm (Xinmingde Machinery, Xinxiang, China) were used for filtration.

Ultrapure water (Barnstead Smart2Pure Water Purification System, Thermo Fisher Scientific, Waltham, MA, USA) was used for all experiments. Before use, all working solutions (distilled water, 10% KOH, and 50% ethanol) were filtered through a 0.22 μm PVDF membrane filter.

### 2.2. Preparation of PET MP BSA-AF488 Hard Corona

To evaluate the efficiency of three different clean-up protocols for removing hard corona from the surface of PET MPs by measuring residual fluorescence, a model system of the hard corona was prepared by incubating PET MPs with BSA-AF488. Briefly, 10 mg of PET MPs (˂80 µm) was mixed with 500 µL of BSA-AF488 working solution (1 mg/mL in simulated intestinal fluid, SIF) in a 2 mL glass vial (Thermo Fisher Scientific, Waltham, MA, USA). The working BSA-AF488 solution was prepared by diluting BSA-AF488 stock solution (5.7 mg/mL) with SIF, which was prepared according to Minekus et al. [17]. The protein/MP ratio in the mixture was 1:20 (*w*/*w*). The reaction mixture was continuously mixed on a rotator (Multi Bio RS-24 Multi-rotator, Biosan, Riga, Latvia) at 37 °C for 4 h. After that, MPs were separated from the supernatant (bulk BSA-AF488 solution) by centrifugation at 1000 rpm for 5 min (Centrifuge 5804 R, Eppendorf, Hamburg, Germany). The pellet of MPs, from which the supernatant was removed, was washed three times with distilled water (1 mL each time) to remove any soft corona from the MPs. For each wash, the pellet was vortexed for 30 s, incubated for 5 min in water, and centrifuged for 2 min at 1000 rpm. After removing the water, the pellet of PET MPs with the formed BSA-AF488 hard corona was subjected to different clean-up protocols.

In this experiment, eight experimental replicates of PET MPs with the formed BSA-AF488 hard corona were prepared. Six replicates were used for three different clean-up protocols (each protocol was repeated in duplicate), and two replicates were used as positive controls (100% fluorescence). Additionally, six experimental replicates of PET MPs incubated with SIF without BSA-AF488 were prepared as negative controls (0% fluorescence) and further subjected to the three different clean-up protocols (each protocol was repeated in duplicate).

### 2.3. Clean-Up Protocols for Removing Adsorbed BSA-AF488 Hard Corona from PET MPs

Three different approaches to the removal of the hard corona of BSA-AF488 from PET MPs were evaluated: the combination of ionic detergent (SDS) with H_2_O_2_ oxidation (protocol I), only oxidation with H_2_O_2_ (protocol II), and the combination of H_2_O_2_ oxidation with an alkali (KOH) (protocol III). Each protocol was performed with two prepared experimental replicates of PET MPs with the formed BSA-AF488 hard corona and negative controls, described in Section 2.2.

#### 2.3.1. Clean-Up Protocol I: The Combination of IONIC Detergent (SDS) and H_2_O_2_ Oxidation

Pellets of PET MPs with the formed BSA-AF488 hard corona that remained in a glass vial were first washed twice with 10% SDS (1 mL each time). Each time, the mixture was continuously mixed on a rotator (Multi Bio RS-24 Multi-rotator, Biosan, Riga, Latvia) at RT for 20 min. After that, MPs were separated from the supernatant by centrifugation at 1000 rpm for 5 min (Centrifuge 5804 R, Eppendorf, Hamburg, Germany). In the next step, MP pellets were washed twice with 1 mL of water. Each time, the pellet was vortexed for 30 s and centrifuged for 2 min at 1000 rpm. After carefully removing water, PET MPs were transferred from the glass vials using a glass pipette onto a vacuum filtration system equipped with a stainless steel filter (mesh size 10 μm, 25 mm diameter) (Xinmingde Machinery, Xinxiang, China). For the transfer of MPs and their further SDS rinsing, 50 mL of water was used. The filter containing PET MPs was transferred into glass beakers, and 30 mL of 15% H_2_O_2_ solution was added. After 30 min of incubation at RT, the samples were sonicated for 1 min in an Elmasonic P30 H ultrasonic bath (Elma Schmidbauer GmbH, Singen, Germany). Finally, the PET MPs were transferred from the beaker onto a new stainless steel filter.

#### 2.3.2. Clean-Up Protocol II: Oxidation with H_2_O_2_

Pellets of PET MPs with the formed BSA-AF488 hard corona remaining in the glass vials were carefully transferred into beakers using a glass pipette and 15 mL of water. After that, 15 mL of 30% H_2_O_2_ was added (the final concentration of H_2_O_2_ was 15%), and the reaction mixture was first incubated for 1 h at RT and then sonicated for 1 min in an Elmasonic P30 H ultrasonic bath (Elma Schmidbauer GmbH, Singen, Germany). PET MPs from the beakers were transferred onto 10 µm stainless steel filters by filtration using a vacuum filtration system. The filters with the PET MPs were returned to the same beakers and covered with 30 mL of 15% H_2_O_2_ and incubated for 1 h at RT. After the sonication of the samples for 1 min in the ultrasonic bath, PET MPs were finally transferred from the beakers onto new stainless steel filters.

#### 2.3.3. Clean-Up Protocol III: The Combination of H_2_O_2_ Oxidation with KOH Digestion

Pellets of PET MPs with the formed BSA-AF488 hard corona remaining in the glass vials were first treated with H_2_O_2_ using the same starting steps described in protocol II. When PET MPs were transferred onto 10 µm stainless steel filters after incubation in the presence of 15% H_2_O_2_ for 1 h at RT and sonication for 1 min, they were returned to the same beaker and covered with 30 mL of 10% KOH. After sonication for 5 min in the ultrasonic bath, the reaction mixture was incubated for 24 h at RT and transferred from the beaker onto new stainless steel filters.

The final step in all three protocols, as well as in the positive control, consisted of rinsing PET MPs three times with 30 mL of water, followed by a single rinse with 30 mL of 50% ethanol. Before subsequent analyses, the filters containing the MPs were placed in a Petri dish and dried at room temperature (RT) in the dark.

### 2.4. Quantification of Residual Fluorescence on PET MPs After Clean-Up Protocols

The clean-up efficiency of each protocol was assessed by the measurement of residual fluorescence on PET MPs using the Synergy LX multi-mode reader (BioTek Instruments; Winooski, VT, USA). Briefly, 2–3 mg of dried PET MP particles from each stainless steel filter was first weighed on a piece of aluminum foil using an analytical balance. In the next step, MP particles were quantitatively transferred using a spatula into the wells of a microtiter plate for fluorescence measurement (Sarstedt, Germany) and suspended in 150 µL of water. Residual fluorescence in the prepared samples and positive and negative controls was measured using a green filter (excitation: 485/20; emission: 528/20) on the Synergy LX multi-mode reader. The fluorescence intensity was first normalized to the mass of the PET MP particles weighed into microtiter wells. Residual fluorescence was calculated according to the following equation: Residual fluorescence of sample (%) = fluorescence intensity of sample × 100/fluorescence intensity of positive control. The positive control (PET MPs with BSA-AF488 hard corona), not subject to clean-up protocols, was considered as the maximal fluorescence (100% intensity). The results were expressed as the mean value ± SD of two measurements for all samples and controls (positive and negative).

### 2.5. Qualitative Analysis of Residual Fluorescence on PET MPs After Clean-Up Protocols

The remaining fluorescence on the PET MPs was also checked qualitatively using reducing SDS polyacrylamide gel electrophoresis (PAGE) followed by fluorescence gel imaging using a Typhoon FLA 7000 imager (GE Healthcare Bio-Sciences AB, Uppsala, Sweden), and images of PET MP particles were taken using an A16.2701 fluorescent microscope (Opto-Edu, Beijing, China).

#### 2.5.1. SDS PAGE and Fluorescence Gel Imaging

After the measurement of residual fluorescence, the suspensions of PET MP particles in the microtiter plate wells were used for the preparation of samples for reducing SDS PAGE. Briefly, the PET MP suspension was quantitatively transferred into 1.5 mL plastic tubes (Eppendorf, Hamburg, Germany) using an automatic pipette, and 37 µL of 5× concentrated reducing buffer for samples was added. The samples were heated at 95 °C for 5 min. A total of 30 µL of each sample (vortexed and centrifugated 5 min 13,000× *g*, Eppendorf, Hamburg, Germany) was loaded into the wells of a 14% gel. After electrophoresis using the Mini-PROTEAN^®^ system (Bio-Rad, Hercules, CA, USA), the gel was scanned using a Typhoon FLA 7000 (GE Healthcare Bio-Sciences AB, Uppsala, Sweden) imager.

#### 2.5.2. Fluorescent Microscopy

After the measurement of dried PET MP particles for the quantification of residual fluorescence, the remaining samples on the filters were used for fluorescence microscopy. One set of MP samples was used for additional staining with Nile red. Briefly, a working solution of Nile red (0.02 mg/mL) was prepared by diluting the stock solution (0.4 mg/mL in acetone) with 50% ethanol. Stainless steel filters containing PET MPs were placed in Petri dishes, and 120 µL of the Nile red working solution was added to cover the samples. After 10 min at RT, an additional 120 µL of the Nile red working solution was added and left for another 10 min. After that, the filters were transferred to a vacuum filtration system, rinsed with 8 mL of 50% ethanol, and left to dry in Petri dishes. Entire filters containing the PET MPs with or without additional staining with Nile red were placed between two microscope slides. To ensure stability during imaging, and the potential loss of MPs, the prepared sandwich was secured with tape on two sides. Each filter was first observed with 4× magnification using a fluorescence microscope (A16.2701, OptoEdu, Beijing, China) and later with 10× magnification. Several microscopic fields of view were captured per sample for each magnification using Image View version 3.7 microscope camera software (Beijing, China) after excitation using UV (340–380 nm) and green (527.5–552.5 nm) excitation filters. All obtained images were not further processed.

### 2.6. ATR FTIR Spectroscopy Analysis of Chemical and Morphological Characteristics of PET MPs

To investigate whether the reagents used in clean-up protocols or formed protein coronas altered the chemical composition and morphological characteristics of MPs and influenced the accurate identification of MP type, the FTIR spectra of PET MPs incubated with or without BSA-AF488 or digestive enzymes after clean-up protocols were compared with naïve PET spectra or with the corresponding spectrum of the control. FTIR spectroscopy was performed using two instruments. Clean-up protocol evaluation was conducted on a Thermo Scientific Nicolet iN10 instrument (Thermo Fisher Scientific, Waltham, MA, USA) equipped with an ATR accessory (Ge crystal) and a liquid nitrogen-cooled MCT detector. Spectra were collected in the 4000–650 cm^−1^ range at 4 cm^−1^ resolution. PET MPs incubated with digestive enzymes were analyzed on a Nicolet Summit with an Everest Diamond ATR, collecting spectra in the 4000–600 cm^−1^ range at 2 cm^−1^ resolution. Data acquisition and processing (normalization and subtraction of spectra) were performed using OMNIC Specta software version 2.2.155 (Thermo Fisher Scientific, Waltham, MA, USA). All spectra were averaged and normalized to a peak at 1408 cm^−1^ prior to spectral overlay or subtraction. The average matching rates (%) between the FTIR spectra obtained for untreated PET particles (native PET MPs) and after their exposure to different clean-up protocols were determined. The chemical identities (% matching) of the FTIR spectra of PET MP samples treated with different clean-up protocols and positive and negative controls with spectra libraries were determined.

### 2.7. Corona Formation on PET MPs with Intestinal Enzymes

To check the influence of intestinal enzymes (chymotrypsin and lipase) on PET MP integrity during the prolonged exposure of plastic to these hydrolytic enzymes, as well as the efficiency of the developed clean-up protocol for the removal of the protein corona, PET MPs were incubated with different concentrations/activities of enzymes in SIF for 24 or 72 h at 37 °C. For this experiment, SIF was prepared according to Minekus et al. [17] with slight modification. NaHCO_3_ was replaced with an equimolar concentration of NaCl to keep the pH more stable during incubation. Physiological enzyme activity was also evaluated based on the same paper [17]. Furthermore, to prevent microbial growth, 0.01% of NaN_3_ was added to SIF for the incubation of lipase for 72 h.

PET MPs were treated with chymotrypsin mimicking a physiological enzyme activity level of 25 IU/mL (one unit hydrolyzes 1 µmole of N-benzoyl-L-tyrosine ethyl ester per minute at pH 7.8 at 25 °C) and incubated for 24 h. To stop digestion after incubation, PMSF to a final concentration of 4.76 mM was added to the samples. In another experiment, PET MPs were exposed to different activity levels of intestinal lipase. MPs were treated with an activity level of lipase of 930 IU/mL (one unit releases 1 µmol of butyric acid per minute at 37 °C and pH 8) for 24 h and a much lower activity level of 75.6 IU/mL for 72 h. This is lower than the proposed physiological lipase activity level for adults, which is 2000 IU/mL [17], but is close to the lipase activity level in infants [18]. Digestion was stopped by either adjusting the pH to 5 when the sample is incubated for 24 h or with the addition of Orlistat to a final concentration of 50 µM. As a control for all experiments, PET MPs were incubated in SIF for the appropriate amount of time. In all experiments, the ratio of MPs to liquid was 1:50.

### 2.8. Statistic

Unpaired *t*-tests were used to compare residual fluorescence intensities after the clean-up protocols, using GraphPad Prism 10.4.2 (GraphPad Software, Boston, MA, USA). Clean-up experiments were performed in duplicate, and data are presented as the mean ± standard deviation (SD). A *p* value of less than 0.05 was considered statistically significant.

## 3. Results

### 3.1. Fluorescence Quantification of Residual Protein on PET MPs

**Figure 1:**
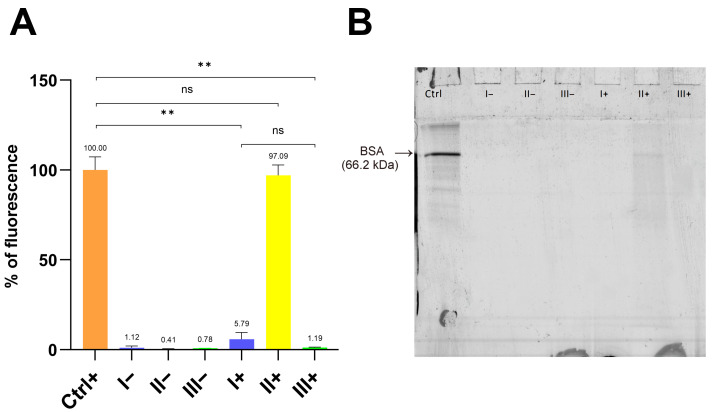
Fluorescence intensity (%) (**A**) and fluorescent image of gel after reducing SDS-PAGE (**B**) of PET MPs with (+) or without (−) BSA-AF488 hard corona following treatment with three clean-up protocols: I (10% SDS + 15% H_2_O_2_); II (2 × 30% H_2_O_2_); III (15% H_2_O_2_ + 10% KOH). Ctrl+—Positive control (PET MPs with BSA-AF488 hard corona without treatment). Fluorescence intensity was normalized to mass of weighed PET MP particles. Then, 30 µL of each sample was loaded into each well. Fluorescence intensity was measured at 485/20 nm excitation and 528/20 nm emission, while fluorescence image was acquired at 473 excitation nm and 520 nm emission. **: *p* < 0.01.

PET MPs, owing to their high surface area and strong binding affinity, readily form protein coronas in environmental media, food matrices, and biological fluids [19,20]. Corona composition and stability depend on protein type and MP physicochemical properties, conferring new environmental and biological identities and leading to outcomes distinct from those of naïve MPs [19]. In this work, we evaluated three clean-up protocols (designated protocols I–III) for removing hard protein coronas from PET MPs formed with fluorescently labeled bovine serum albumin (BSA-AF488) under simulated intestinal fluid conditions. The efficiency of removing the PET MP hard corona using the developed protocols was first evaluated by quantifying residual fluorescence (Figure 1A).

The application of clean-up protocols I and III—based on 10% SDS/15% H_2_O_2_, and 15% H_2_O_2_/10% KOH, respectively—resulted in a statistically significant (*p* < 0.01) reduction in fluorescence intensity on PET MPs compared with the positive control (PET MPs with BSA-AF488 hard corona). Although the residual fluorescence after protocol I was implemented (5.79 ± 3.94%) was higher than that after protocol III (1.19 ± 0.30%), the difference was not statistically significant. Protocol II, which employed only H_2_O_2_ (2 × 15% treatments), yielded the highest residual fluorescence (97.06 ± 6.08%) and did not differ significantly from the positive control, indicating the poor removal of the BSA hard corona. Negative controls (PET MPs without BSA-AF488, subjected to clean-up with protocols I–III) showed residual fluorescence values ranging from 0.41 ± 0.11% to 1.18 ± 0.99%.

Overall, the results indicate that clean-up protocols combining an ionic detergent with H_2_O_2_ (protocol I) or H_2_O_2_ with KOH (protocol III) are sufficiently effective for the near-complete removal of BSA hard coronas from PET MPs.

### 3.2. SDS-PAGE Analysis of Protein Residues on PET MPs

To validate the efficiency of protocols I–III in removing the BSA-AF488 hard corona, SDS-PAGE was performed using the same samples that were analyzed for residual fluorescence (Figure 1B). After the excitation of the gel at 473 nm, the strongest fluorescent band from BSA-AF488 was observed for the positive control samples (Ctrl+). Additionally, a very weak band at the same position was observed in PET MPs treated with protocol II (2 × 15% H_2_O_2_), whereas no fluorescence bands were detected in the other samples.

These results indicate that BSA-AF488 bands remained only after treating PET MPs with H_2_O_2_ alone. Comparing the results of both experiments, the bands in the gel for the positive control and protocol II+ should be of similar intensity. The lower intensity of the band for sample II+ compared to the positive control can be explained by the fact that residual fluorescence in the first experiment was expressed following normalization to the mass of the weighed PET MP particles. The measured particle masses for the positive control and II+ samples were 2.67 mg and 2.19 mg, respectively, suggesting that the expected fluorescence for sample II+ would be at least 22% lower than in the positive control.

Another possible factor contributing to the lower band intensity of the protocol II+ sample after SDS-PAGE, compared with the positive control, is the difference in the thickness of the BSA-AF488 coronas. This can be explained by the fact that BSA is an ellipsoid, with a 4 × 4 × 8.3 nm size at pH 4–9 and an asymmetric charge distribution. Depending on the surface characteristics of the material, BSA can adsorb in a side-on or end-on orientation or form a multilayer film [21]. To our knowledge, no data are available on the morphology of BSA coronas on PET MPs. To date, polystyrene (PS) nanoplastics (NPs), widely used as model systems for medical nanocarriers, have been the most common plastic type investigated for BSA coronas. References [22,23,24] reported that serum proteins formed a ~70–100 nm thick soft protein corona on PS NPs. After three washing and centrifugation steps, the remaining hard corona had a thickness of approximately 15 nm, as measured by transmission electron microscopy. They also found that human serum albumin (HSA) was the most abundant protein in the soft corona (42% of total identified proteins), whereas its abundance in the hard corona was only 3%. Given that naïve PS and PET have a similar surface hydrophobicity and charge, that BSA and HSA share the same properties, and that three washing steps were used for hard corona preparation, we conclude that BSA-AF488 likely formed a very thin hard corona in the positive control. Treatment with protocol II may remove some BSA-AF488 molecules from the outer layers of the BSA corona, resulting in a lower total quantity of BSA molecules and reduced fluorescence intensity compared with the positive control, as observed in the SDS-PAGE analysis. Conversely, if the BSA hard corona in the positive control consists of several layers, the BSA-AF488 molecules in the inner layers may not be excited during fluorescence intensity measurements, leading to an underestimation of the actual fluorescence. Consequently, the positive control and the II+ sample exhibited similar residual fluorescence values (Figure 1A).

### 3.3. Microscopy-Based Assessment of Hard Corona of MPs

**Figure 2:**
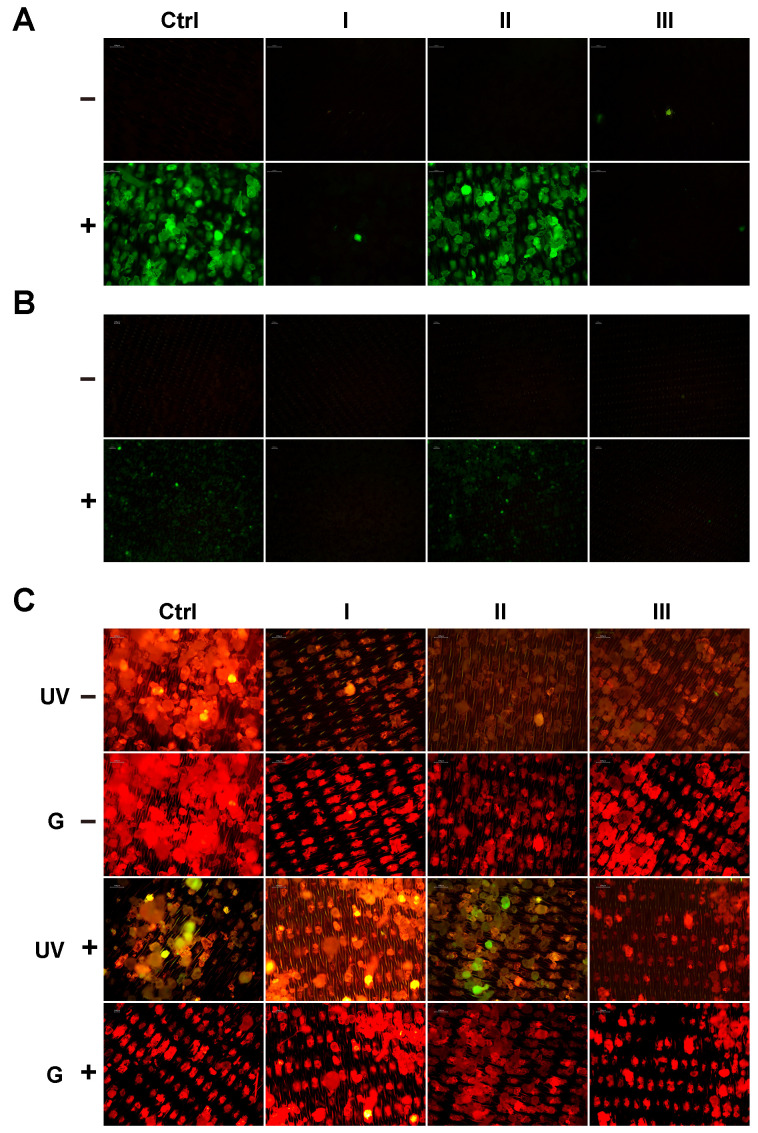
Fluorescence images of PET MPs with (Ctrl+) or without (Ctrl−) BSA-AF488 hard corona acquired with 10× (**A**) and 4× magnification (**B**) and after staining with Nile red (**C**) following treatment with three clean-up protocols: I (10% SDS + 15% H_2_O_2_); II (2 × 30% H_2_O_2_); III (15% H_2_O_2_ + 10% KOH). Ctrl+ and Ctrl−: Positive and negative control (PET MPs with or without BSA-AF488 hard corona without any treatments). Images were acquired with 10× magnification after excitation at 340–380 nm (UV filter for BSA-AF488) and/or 527.5–552.5 nm (G—green filter for Nile red).

To evaluate visual differences between hard coronas after clean-up protocols, fluorescence images were acquired for PET MPs with or without a BSA-AF488 hard corona (Figure 2A,B). Additional staining with Nile red was performed (Figure 2C), and images were captured at 10× (Figure 2) and 4× magnification (Supplementary Section S1, Figure S1).

Upon the excitation of BSA-AF488 using a UV filter, the positive control and II+ samples showed strong green fluorescence from all PET MP particles (Figure 2A). In contrast, in all negative controls and after treatment with protocols I and III, fluorescence was observed only for a few individual particles, which was consistent with previous experimental results.

PET MPs, with or without a BSA-AF488 hard corona, were additionally stained with Nile red. All samples exhibited strong red fluorescence under green filter excitation. Under UV excitation, fluorescence emission varied by sample, appearing green, orange, or red (Figure 2C). In the positive control and II+ samples, some particles displayed exclusively green or orange emission, while many showed intermediate hues. I+ and III+ samples emitted predominantly orange and yellow fluorescence, respectively. Nile red staining revealed that the BSA-AF488 hard corona morphology was heterogeneous across PET particles.

Nile red is an uncharged hydrophobic dye whose fluorescence is strongly influenced by the polarity of its surrounding environment. In non-polar environments, such as hydrophobic lipids or plastics, Nile red exhibits emission in the yellow-orange region. In moderately polar environments, such as lipid–protein mixtures or biological membranes, its emission shifts to the red-orange region [25]. Nile red can also interact with a variety of native proteins, including β-lactoglobulin, κ-casein, and albumin, producing a wide range of spectral shifts depending on the protein [25]. The different fluorescence colors observed in the positive control and II+ samples confirm that BSA-AF488 and Nile red can bind to the same particle. Depending on the structure of the formed hard corona, the ratio of bound BSA-AF488 to Nile red molecules per particle can vary, resulting in differences in emitted color. In this study, PET MPs were prepared by milling, producing particles with a broader range of shapes, sizes [16], and surface roughness compared with PS NPs prepared by solvent precipitation [24]. The amount of protein incorporated into the hard corona is influenced by the roughness of the surface. Previous studies have reported that particles with smoother surfaces adsorb more BSA molecules than those with rougher surfaces [26,27].

### 3.4. ATR FTIR Analysis of PET Integrity After Clean-Up Protocols

**Figure 3:**
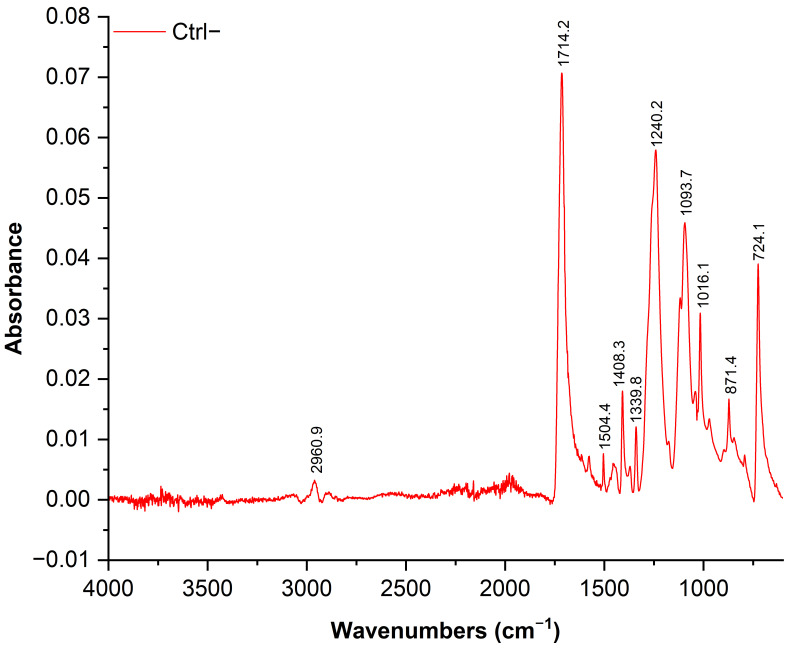
ATR-FTIR spectrum of untreated PET MPs (Ctrl−).

To assess the influence of three clean-up protocols on PET integrity, we focused on six major characteristic FTIR absorption bands corresponding to the stretching of the ester carbonyl group ν(C=O), located at 1710–1725 cm^−1^; the aromatic ring C-H in-plane deformation δ(C-H_in_) at 1408 cm^−1^; the wagging of the methylene group w(CH_2_) at 1340 cm^−1^; the stretching of the ester bond ν(C=O)-O, at 1240 cm^−1^; the stretching of the glycolic bond ν(O-CH_2_), at 1094 cm^−1^; and the out-of-plane C-H deformation of the aromatic ring δ(C-H_out_), at 724 cm^−1^ [28]. These bands were chosen in order to characterize degradation or hydrolysis [29,30]. Additionally, aliphatic C-H bond stretching at 2960 cm^−1^ was present as well (Figure 3). Sharp peaks at ~1714 cm^−1^ and 1240 cm^−1^ suggest no chemical (hydrolysis) nor physical changes in the PET MP used in this study.

**Figure 4:**
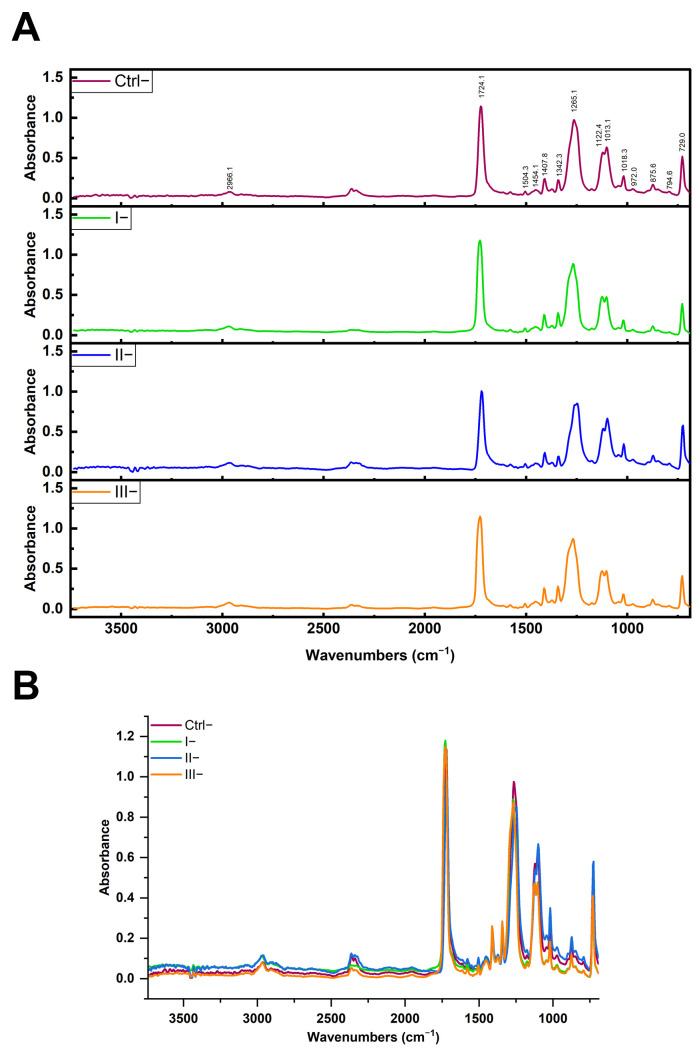
ATR-FTIR spectra (**A**) and overlaid spectra (**B**) of PET MPs incubated without BSA-AF488 and treated with different clean-up protocols I (10% SDS + 15% H_2_O_2_), II (2 × 15% H_2_O_2_), and III (15% H_2_O_2_ + 10% KOH), compared to untreated PET MPs (Ctrl−).

To verify whether the reagents applied in the clean-up protocols affected the native PET MPs, we conducted ATR-FTIR analysis on untreated PET MPs and those treated with protocols I (10% SDS + 15% H_2_O), II (2 × 15% H_2_O_2_), and III (15% H_2_O_2_ + 10% KOH) (Figure 4A). ATR-FTIR was chosen due to its higher surface sensitivity [31]. In the ATR-FTIR spectra of PET MP samples treated with SDS, H_2_O_2_, or KOH, the characteristic vibrational bands of the PET polymer appeared at essentially the same wavenumbers as in the untreated PET sample. In particular, the key PET peaks, ~1720 cm^−1^, 1400–1500 cm^−1^, and 1100–1250 cm^−1^, were observed at unchanged positions (Figure 4B). Only minor variations in band intensity were noted (likely due to surface cleanliness), but no new peaks appeared, and no peak shifts were detected. A comparison of the obtained spectra with untreated PET MPs (Ctrl−) (Table 1) allowed us to confirm that none of the cleaning protocols induced significant changes in the characteristic absorption bands of PET (correlation values between spectra were 0.978–0.988), indicating the preservation of polymer chemical integrity throughout the cleaning processes.

### 3.5. Residual Protein Presence Evaluation on PET MPs

Beyond polymer characterization, FTIR can also provide insight into residual protein presence on plastic surfaces. Proteins typically exhibit distinct absorption bands in the infrared spectrum, including the amide I band (~1650 cm^−1^), which arises primarily from the C=O stretching vibrations of the peptide backbone, and the amide II band (~1540 cm^−1^), which is attributed to N–H bending and C–N stretching [32]. The intensity and position of these bands can vary depending on protein conformation and interactions with surfaces [33]. ATR-FTIR analysis was performed to evaluate potential spectral changes on the surface of PET MPs after incubation with BSA-AF488 due to residual protein presence or protein–plastic interactions.

**Figure 5:**
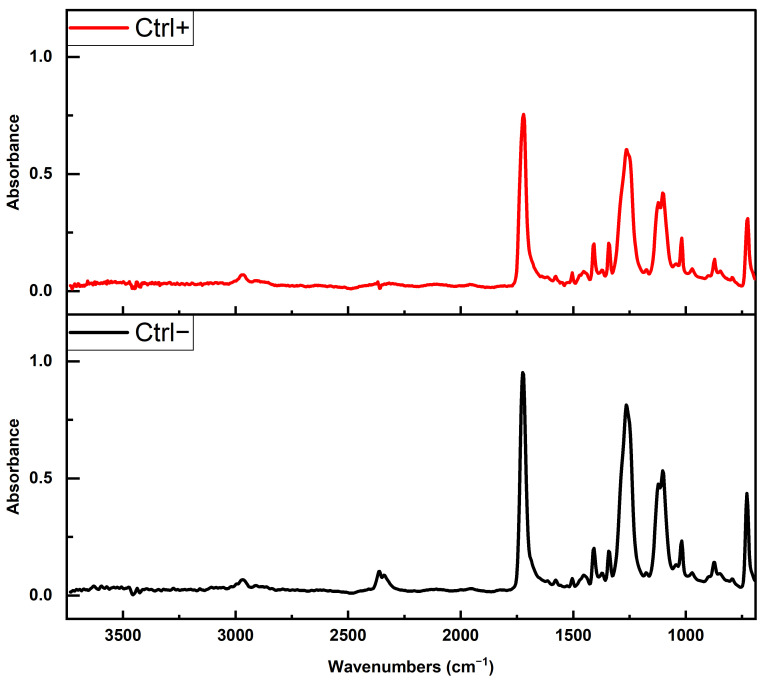
ATR-FTIR spectra of PET MPs with BSA-AF488 hard corona compared to untreated PET MPs.

The spectrum of the PET MPs with the BSA-AF488 hard corona (Ctrl+) showed a high similarity with the untreated PET MPs (Ctrl−), with a correlation coefficient of 0.988 (Figure 5, Supplementary Section S2, Figure S2). No distinct new absorption bands were detected in the amide I and II region. The absence of a discernible amide I band is likely attributable to an overlap with the intense ester carbonyl absorption of PET, observed near ~1715 cm^−1^. The amide I and II bands, commonly employed for protein secondary structure analysis, were not detected in our PET MP sample with the BSA-AF488 hard corona. Also, this absence can be attributed to the thin and uneven distribution of the protein corona on the MP surface, as revealed by Nile red staining (Figure 2C), which likely limits its detectability by ATR-FTIR spectroscopy [32].

Subtle changes could be seen in subtraction spectra (Figure S2). A shift in the band at 1717 cm^−1^ points to the changed polarity around the ester bond due to protein binding. The decreased intensity in the region of 1200 cm^−1^ to 900 cm^−1^ may be because of protein binding but without signatures of amide I or II bands in PET MP with the BSA-AF488 hard corona sample.

**Figure 6:**
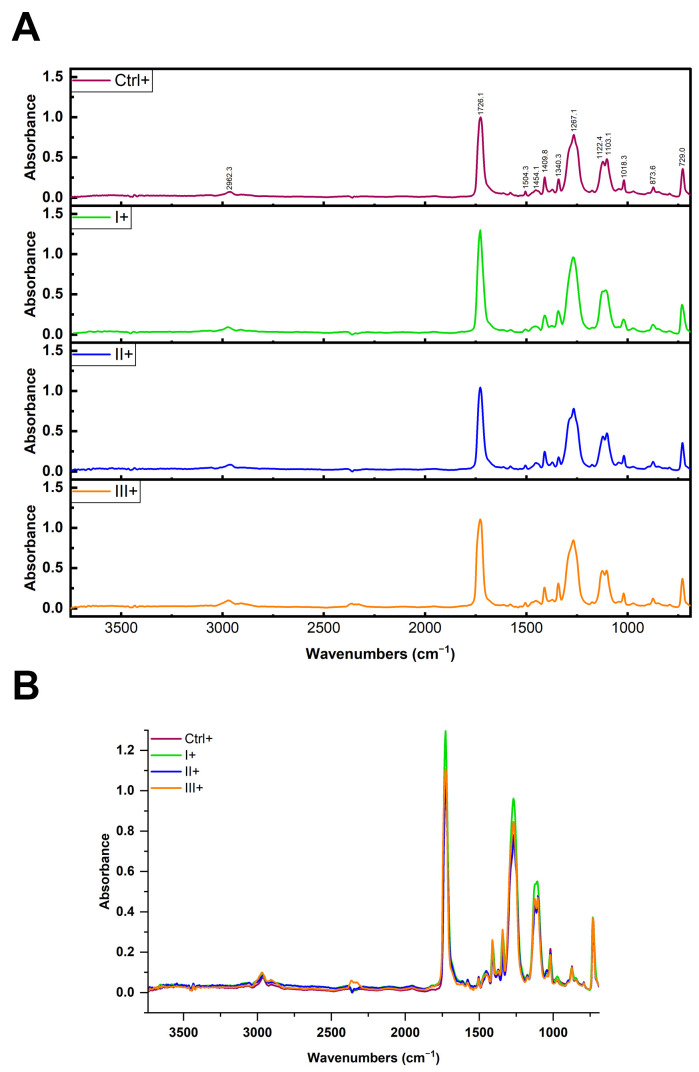
ATR-FTIR spectra (**A**) and overlaid spectra (**B**) of PET MPs incubated with BSA-AF 488 and treated with different clean-up protocols I+ (10% SDS + 15% H_2_O), II+ (2 × 15% H_2_O_2_), and III (15% H_2_O_2_ + 10% KOH), compared to positive control (PET MPs incubated with BSA-AF488 with hard corona, Ctrl+).

ATR-FTIR analysis was also performed to evaluate potential spectral changes on the surface of PET MPs after incubation with BSA-AF488 and treatment with different clean-up protocols (Figure 6). In all samples, the characteristic vibrational bands of the PET polymer appeared at essentially the same wavenumbers as in the positive control, without the emergence of new peaks or shifts in existing peaks (Figure 6B) and with only minor variations in band intensity, likely attributable to differences in surface cleanliness. No clear amide I and II bands were detected in any of the samples, which suggests more efficient protein removal. Furthermore, no traces of SDS were detected in the sample in which protein removal was performed with ionic detergent. These results indicate that the cleaning treatments removed external contaminants without chemically modifying the PET polymer.

This absence of protein-specific signals is consistent with the fluorescence and SDS-PAGE data, supporting the conclusion that all cleaning protocols were efficient in the removal of bulk proteins. The remaining hard corona proteins (especially after the protocol applying only oxidative treatment) did not affect PET spectral characteristics. All protocols preserved the key spectral features of PET, indicating that no significant chemical degradation occurred, even under strong alkaline or oxidative conditions, as we reported in a previous study [34]. Furthermore, the less than optimal removal of the protein corona by oxidative treatment did not affect polymer identification and characterization by FTIR.

### 3.6. ATR-FTIR Analysis of PET MPs After Digestive Enzyme Exposure

To further evaluate corona properties in the presence of digestive enzymes, chymotrypsin, lipase, and trypsin were incubated with PET in SIF for 24 or 72 h at 37 °C to allow for corona formation. After digestion, enzyme activity was inhibited, and clean-up protocols effective for removing the hard corona of BSA, based on SDS + H_2_O_2_ or H_2_O_2_ + KOH, were applied. ATR-FTIR spectra were analyzed to assess protein–polymer interactions and to detect potential spectral changes induced by the combined effect of enzymatic activity and clean-up protocols. Incubation times longer than physiological times were tested, as previous studies reported that the exposure of PET MPs to pancreatin under physiological conditions resulted in only minimal changes, as determined by micro-Raman analysis [11].

**Figure 7:**
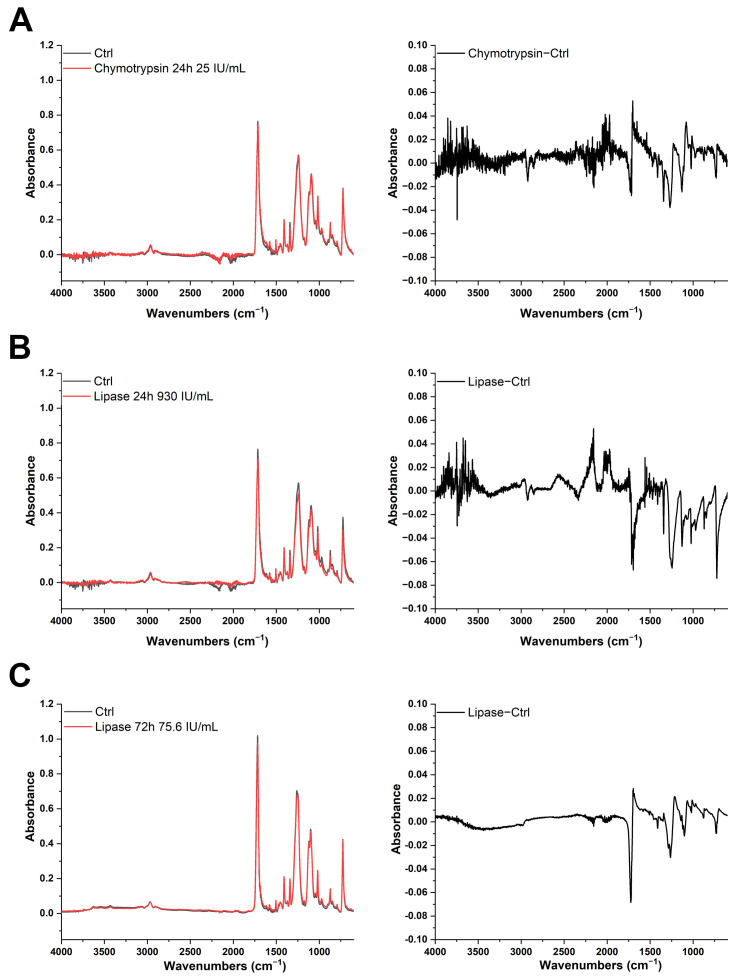
Overlaid spectra (**left**) and difference spectra (**right**) of PET MPs incubated for 24 h with chymotrypsin 25 IU/mL (**A**), 24 h with lipase 930 IU/mL (**B**), and 72 h with lipase 75.6 IU/mL in simulated intestinal fluid and corresponding controls (**C**) (PET MPs incubated without enzymes under same conditions). Clean-up protocol based on H_2_O_2_ + KOH was used to remove hard and soft coronas.

All spectra were comparatively analyzed across multiple conditions such as the time of incubation, enzyme activity, and clean-up protocols (Figure 7, Supplementary Section S3.1, Figure S3). A comprehensive analysis of the obtained spectra revealed no significant differences in the spectral profiles between the enzyme-treated and control samples under the applied conditions (Figure 7 and Figure S3, left panel). The characteristic absorbance bands of PET, including the carbonyl stretch (C=O, 1717 cm^−1^), aromatic ring vibrations (1600–1400 cm^−1^), and ester stretches (asymmetric and symmetric C-O, 1260 and 1099 cm^−1^, respectively), remained unchanged throughout the experiment. All these absorption bands associated with PET were consistently observed in all samples. Importantly, no new functional group signals emerged, and PET’s core chemical structure remained intact across treated samples, indicating that the enzymatic treatment did not induce any chemical modifications detectable by FTIR spectroscopy. Furthermore, no protein-associated peaks (amide I and amide II bands) could be observed after the applied protocols. This supports and further extends previous findings that PET is resistant to hydrolytic or enzymatic degradation under physiologically relevant conditions [35], even in the case of longer than physiological incubation with digestive fluids and enzymes.

Subtle changes could be observed upon PET MP incubation with lipase for 24 h and 72 h (Figure 7B,C, right panel). There is a decrease in peak intensity at 720 cm^−1^ (characteristic of PET crystallinity, suggesting the disruption of the crystalline structure [36]) and a decrease and broadening of the 1717 cm^−1^ C=O peak, without chemical transformation occurring, as well as a decrease in a peak at 1093 cm^−1^ and 1260 cm^−1^. All these changes strongly suggest slight physical changes in PET upon prolonged incubation with lipase using lower activity levels (75.6 and 930 IU/mL) in longer incubation periods.

Finally, as the longer incubation period (up to 72 h) at 37 °C may be optimal for microbial growth (air contamination or introduced with MPs), incubation mixtures were analyzed for sterility (Supplementary Section S3.3, Figure S5). No contamination occurred during the 72 h lipase (Figure 7C) incubation due to the addition of sodium azide.

No protein residues, even in the case of microbial biofilm formation, were detected in any of the samples after applying cleaning protocols (SDS/H_2_O_2_ and H_2_O_2_/KOH), indicating their efficacy.

## 4. Discussion

The results of this study provide insights into the mechanistic underpinnings of protein–microplastic (MP) interactions and the stability of the hard protein coronas formed in simulated intestinal fluid conditions. Using BSA as a model protein and PET MPs under intestinally simulated conditions, we demonstrated that corona formation is not only robust but also resistant to disruption by oxidative treatment alone. These findings underscore the complexity of protein adsorption mechanisms on MP surfaces and the importance of selecting appropriate decontamination strategies in analytical workflows.

Our previous study showed that oxidative treatment with 15% H_2_O_2_ was less effective than digestion with 10% KOH for removing organic matter during the isolation of MPs from seafood samples [34]. The development of new protocols that enable the investigation of eco- and bio-coronas of various types of MPs is essential for a better understanding of the interactions between coronas and MPs. Because the SDS/H_2_O_2_ protocol is less time-consuming and uses less aggressive reagents, it may be suitable for the rapid removal of hard coronas from various types of MPs. Given that environmental plastic pollution is one of the most critical environmental and toxicological issues [37,38], identifying natural resources capable of degrading plastics and elucidating the toxicity of MPs coronas are still major challenges for scientists. Currently, the methods used for the isolation of MPs from environmental and biological samples often involve prolonged exposure to aggressive reagents such as an acid or alkali, which can lead to the loss of eco- and bio-coronas but also the plastic itself [39]. This may lead to a misinterpretation of the results concerning plastic integrity preservation, particularly when the primary aim of the investigation is to assess the influence of eco- and bio-coronas on plastic integrity.

In the current study, we observed no measurable changes to PET’s key FTIR spectral markers following treatment, indicating that none of the protocols, oxidative, alkaline, or surfactant-based, compromised polymer integrity. In our spectra, all PET samples maintained these distinct peaks regardless of treatment, indicating that no significant polymer chain cleavage or oxidation occurred. This confirms that none of the clean-up protocols, including the SDS/H_2_O_2_ or H_2_O_2_/KOH combination, adversely affected the chemical structure of PET MPs.

Among the tested protocols, the combination of SDS/H_2_O_2_ or H_2_O_2_/KOH achieved the most effective removal of the protein corona. This observation suggests that corona stability is maintained by a combination of hydrophobic and ionic interactions that require both surfactant-mediated disruption and alkaline hydrolysis for complete dissociation. The poor performance of hydrogen peroxide treatment implies that oxidative cleavage, valuable for general organic digestion, may not sufficiently disrupt the cohesive forces anchoring the hard corona to the PET surface.

SDS-PAGE and fluorescence quantification both supported the conclusion that significant fractions of BSA remained bound following only oxidative treatment. Moreover, FTIR analysis confirmed that none of the clean-up protocols visibly degraded the PET polymer, validating their use for subsequent spectroscopic analyses. Several recent studies have addressed the challenge of organic matter removal from MP surfaces prior to FTIR spectroscopy, particularly in cases of studying fine structural changes during the biodegradation of plastic [30]. Furthermore, residual proteins remaining on the plastic may obscure or mimic spectral shifts occurring during biotransformation.

Therefore, it was particularly relevant to observe that even the partial removal of the protein corona (such as using only hydrogen peroxide) did not cause significant changes in the FTIR spectra of PET. Even though a layer of hard corona protein remained bound to the MP, it did not contribute to the signal recording by FTIR.

A growing body of work has also examined the behavior of MPs in digestive environments, particularly within intestinal fluids. Recent studies show that MPs incubated in simulated intestinal fluids form stable bio-coronas composed of bile salts and digestive enzymes, altering their colloidal stability and cellular uptake [3,40]. Similarly, Brower et al. reported that MPs exposed to intestinal environments exhibit protein adsorption that persists despite digestion, supporting the notion of resilient hard corona formation [41].

Similarly, protein corona formation on polystyrene nanoparticles has been investigated under gastrointestinal conditions, revealing that digestive proteins can form resilient complexes that modulate particle bioactivity [42]. Other authors used molecular modeling to show that plastic–protein interactions are dominated by hydrophobic forces, supporting our interpretation of hydrogen peroxide’s partial efficacy and the enhanced disruption achieved by SDS + H_2_O_2_ or H_2_O_2_ + KOH [43].

Therefore, our data contributes not only to methodological refinement but also to a broader understanding of corona dynamics in real-world matrices, where hard protein coronas are known to form irreversible complexes that modulate surface properties and bioactivity. In the context of MPs, such persistent coronas may lead to the underestimation of particle numbers, misidentification of polymer types, or misinterpretation of surface properties in environmental and toxicological studies.

These results emphasize that the effective clean-up of MP-associated biomolecules requires a mechanistic understanding of corona formation and persistence. The SDS/H_2_O_2_ or H_2_O_2_/KOH protocol offers a practical, polymer-compatible strategy for improving MP analysis, particularly in biological matrices where protein adsorption can obscure detection and characterization. This work also contributes to broader efforts in standardizing MP isolation methods and deepens our understanding of MP–biomolecule interactions in realistic exposure scenarios.

The use of a single protein for protocol efficacy assessment gave us valuable information on the limitations of our detection method, e.g., the potential interference of the residual protein corona on the detection of subtle peak shifts. However, complex mixtures require further evaluation, as multiple proteins exhibit different affinities towards MPs and compete for a place in the hard corona [20]. This is further complicated when all components of the digestive tract are taken into account, such as bile salts and the microbiome.

Prior studies on the gastrointestinal transformation of PET assessed by micro-Raman have investigated pancreatin digestion (a mixture of hydrolytic enzymes and bile acids) and also looked into microbiome transformations. The conditions tested were considered physiological (2 h incubation in the intestinal fluid) [11]. Subtle morphological changes were observed with intestinal conditions (pancreatin), and they were more dramatic with microbial (colon) transformation. Our study did not apply physiological conditions but applied longer incubation times with individual enzymes to pinpoint the hydrolytic enzymes that may be responsible for mild PET MP surface modifications among pancreatin components observed by Tamargo et al. [11].

Overall, the FTIR spectral analysis reveals enzyme-specific interactions with PET MPs, with lipase exhibiting the most pronounced effects. Together with cutinase and hydrolase, lipase is one of the common enzymes associated with plastic degradation. Lipases have been produced in many bacterial and fungal strains. Lipase is one of the best biocatalysts for PET degradation. Lipase B (CALB) from the yeast *Candida antarctica* is known for its high selectivity and catalytic activity [44]. CALB demonstrated high-efficiency hydrolysis steps and polymer scission that led to the accumulation of terephthalic acid [45].

Intestinal lipase, a component of pancreatin, appears to induce the local hydrolysis of ester bonds, creating structural irregularities and disrupting crystalline regions, hence amorphization. This process increases polymer surface area and accessibility, reduces physical stability, and may precede or accompany biodegradation by the microbiome in the colon. The increased amorphization of PET may be a consequence of the plasticizing effect of polar solvent penetration into PET MP, which breaks hydrogen bonds and disrupts crystallinity. Any changes in crystallinity occurring because of MP exposure to enzymatic action (esterase, i.e., cutinase, lipase, PETase) occurring internally may be considered as internal aging or the weathering of the MP. This process is subtle but can facilitate the further action of the microbiome on the MP in the colon by providing more anchoring points for biofilm formation.

## 5. Conclusions

This study provides an evaluation of protein corona stability and removal from PET MPs under simulated intestinal conditions. Using a combination of fluorescence, protein assays, and FTIR spectroscopy, we demonstrate that PET forms a persistent, thin, and uneven hard corona with BSA, whose nearly complete removal requires either a surfactant combined with oxidation or oxidation followed by strong alkaline treatment. Additionally, none of the cleaning protocols caused significant changes in the characteristic absorption bands of PET, indicating that the polymer’s chemical integrity was preserved throughout the cleaning process. Furthermore, among the tested digestive enzymes, only lipase induced subtle spectral changes indicative of surface amorphization, while chymotrypsin and trypsin showed minimal interaction. Moreover, the cleaning treatments can remove not only hard coronas of digestive enzymes but also external contaminants such as microorganisms, without chemically modifying the PET polymer. Together, these data indicate that protein corona removal is not merely a function of chemical strength but requires tailored strategies that reflect the physicochemical characteristics of both the MP surface and the adsorbed protein layer.

## Figures and Tables

**Table 1:** Comparison (expressed as correlation value) between spectra of PET MP samples incubated without BSA-AF488 and treated with clean-up protocols I (10% SDS + 15% H_2_O_2_), II (2 × 15% H_2_O_2_), and III (15% H_2_O_2_ + 10% KOH) and untreated PET MPs.

Protocol	Correlation Value *
I	0.988
II	0.983
III	0.978

* 1 = perfect match; 0 = no match.

## Data Availability

The original contributions presented in the study are included in the article/Supplementary Materials. Further inquiries can be directed to the corresponding author.

## Supplementary Materials

The following supporting information can be downloaded at: https://www.mdpi.com/article/10.3390/foods14203454/s1, Figure S1. Fluorescence images of PET MPs with (Ctrl+) or without (Ctrl−) BSA-AF488 hard corona after staining with Nile red following treatment with three clean-up protocols: I (10% SDS + 15% H2O2); II (2 × 30% H_2_O_2_); III (15% H_2_O_2_ + 10% KOH).  Figure S2. Overlaid spectra (A) and comparison of difference spectra (B) of PET MPs with BSA-AF488 hard corona (Ctrl+) and untreated PET MPs (Ctrl−). Figure S3. Overlaid spectra (left) and the difference spectra (right) of PET MPs incubated: 72 h with chymotrypsin 50 IU/mL (A), 72 h with trypsin 200 IU/mL (B) and 24 h with lipase 4700 IU/mL (C) in simulated intestinal fluid and corresponding controls (PET MPs incubated without enzymes under the same conditions). Clean-up protocols based on SDS + H_2_O_2_ for chymotrypsin and trypsin and protocol based on H_2_O_2_ + KOH were used for removing hard and soft coronas. Figure S4. SDS-PAGE of digestive enzymes used in the experiments on 14% polyacrylamide gel. Mw—molecular weight marker; T—trypsin; C—chymotrypsin; L—lipase. Figure S5. Testing of microbial growth for PET MP samples incubated with intestinal enzymes. A—SIF after 24 h; B—25 U/mL chymotrypsin after 24 h; C—930 U/mL lipase after 24 h; D—SIF after 72 h; E—50 U/mL chymotrypsin after 72 h; F—200 U/mL trypsin after 72 h.

## References

[1] Siddiqui S.A., Bahmid N.A., Salman S.H.M., Nawaz A., Walayat N., Shekhawat G.K., Gvozdenko A.A., Blinov A.V., Nagdalian A.A., Özogul F. (2023). Chapter Eight—Migration of microplastics from plastic packaging into foods and its potential threats on human health. Advances in Food and Nutrition Research.

[2] Rafa N., Ahmed B., Zohora F., Bakya J., Ahmed S., Ahmed S.F., Mofijur M., Chowdhury A.A., Almomani F. (2024). Microplastics as carriers of toxic pollutants: Source, transport, and toxicological effects. Environ. Pollut..

[3] Stock V., Böhmert L., Lisicki E., Block R., Cara-Carmona J., Pack L.K., Selb R., Lichtenstein D., Voss L., Henderson C.J. (2019). Uptake and effects of orally ingested polystyrene microplastic particles in vitro and in vivo. Arch. Toxicol..

[4] Schöpfer L., Schnepf U., Marhan S., Brümmer F., Kandeler E., Pagel H. (2022). Hydrolyzable microplastics in soil—Low biodegradation but formation of a specific microbial habitat?. Biol. Fertil. Soils.

[5] Zhang Q., Xu E.G., Li J., Chen Q., Ma L., Zeng E.Y., Shi H. (2020). A Review of Microplastics in Table Salt, Drinking Water, and Air: Direct Human Exposure. Environ. Sci. Technol..

[6] Elmer-Dixon M.M., Fawcett L.P., Sorensen E.N., Maurer-Jones M.A. (2024). Bovine Serum Albumin Bends Over Backward to Interact with Aged Plastics: A Model for Understanding Protein Attachment to Plastic Debris. Environ. Sci. Technol..

[7] Givens B.E., Diklich N.D., Fiegel J., Grassian V.H. (2017). Adsorption of bovine serum albumin on silicon dioxide nanoparticles: Impact of pH on nanoparticle–protein interactions. Biointerphases.

[8] Buchholz P.C., Feuerriegel G., Zhang H., Perez-Garcia P., Nover L.L., Chow J., Streit W.R., Pleiss J. (2022). Plastics degradation by hydrolytic enzymes: The plastics-active enzymes database—PAZy. Proteins Struct. Funct. Bioinform..

[9] Magalhães R.P., Cunha J.M., Sousa S.F. (2021). Perspectives on the role of enzymatic biocatalysis for the degradation of plastic PET. Int. J. Mol. Sci..

[10] Lowe M.E. (1997). Structure and function of pancreatic lipase and colipase. Annu. Rev. Nutr..

[11] Tamargo A., Molinero N., Reinosa J.J., Alcolea-Rodriguez V., Portela R., Bañares M.A., Fernández J.F., Moreno-Arribas M.V. (2022). PET microplastics affect human gut microbiota communities during simulated gastrointestinal digestion, first evidence of plausible polymer biodegradation during human digestion. Sci. Rep..

[12] Sofield C.E., Anderton R.S., Anyaegbu C.C., Chiu L.S., Fuller K.A., Gorecki A.M. (2025). Digestion of Microplastics with Simulated Gastrointestinal Conditions Mitigates Uptake by Intestinal Epithelial Cells: Quantified by Imaging Flow Cytometry. J. Hazard. Mater..

[13] Kihara S., Ghosh S., McDougall D.R., Whitten A.E., Mata J.P., Köper I., McGillivray D.J. (2020). Structure of soft and hard protein corona around polystyrene nanoplastics—Particle size and protein types. Biointerphases.

[14] Dawson A.L., Bose U., Ni D., Nelis J.L.D. (2024). Unravelling protein corona formation on pristine and leached microplastics. Microplast. Nanoplast..

[15] Jasinski J., Wilde M.V., Voelkl M., Jérôme V., Frohlich T., Freitag R., Scheibel T. (2022). Tailor-made protein corona formation on polystyrene microparticles and its effect on epithelial cell uptake. ACS Appl. Mater. Interfaces.

[16] Lujic T., Gligorijevic N., Stanic-Vucinic D., Krstic Ristivojevic M., Mutic T., Wimmer L., Dailey L.A., Cirkovic Velickovic T. (2025). Effects of Polypropylene and Polyethylene Terephthalate Microplastics on Trypsin Structure and Function. Int. J. Mol. Sci..

[17] Minekus M., Alminger M., Alvito P., Ballance S., Bohn T., Bourlieu C., Carrière F., Boutrou R., Corredig M., Dupont D. (2014). A standardised static in vitro digestion method suitable for food–an international consensus. Food Funct..

[18] Ménard O., Bourlieu C., De Oliveira S.C., Dellarosa N., Laghi L., Carrière F., Capozzi F., Dupont D., Deglaire A. (2018). A first step towards a consensus static in vitro model for simulating full-term infant digestion. Food Chem..

[19] Cao J., Yang Q., Jiang J., Dalu T., Kadushkin A., Singh J., Fakhrullin R., Wang F., Cai X., Li R. (2022). Coronas of micro/nano plastics: A key determinant in their risk assessments. Part. Fibre Toxicol..

[20] Kaseke T., Jovanovic V., Wimmer L., Vasovic T., Mutic T., Acimovic J., Dailey L.A., Velickovic T.C. (2025). Polypropylene micro-and nanoplastics affect the digestion of cow’s milk proteins in infant model of gastric digestion. Environ. Pollut..

[21] Jachimska B., Pajor A. (2012). Physico-chemical characterization of bovine serum albumin in solution and as deposited on surfaces. Bioelectrochemistry.

[22] Luo H., Du Q., Zhong Z., Xu Y., Peng J. (2022). Protein-coated microplastics corona complex: An underestimated risk of microplastics. Sci. Total Environ..

[23] Dong Z., Hou Y., Han W., Liu M., Wang J., Qiu Y. (2020). Protein corona-mediated transport of nanoplastics in seawater-saturated porous media. Water Res..

[24] Kokkinopoulou M., Simon J., Landfester K., Mailänder V., Lieberwirth I. (2017). Visualization of the protein corona: Towards a biomolecular understanding of nanoparticle-cell-interactions. Nanoscale.

[25] Sackett D.L., Wolff J. (1987). Nile red as a polarity-sensitive fluorescent probe of hydrophobic protein surfaces. Anal. Biochem..

[26] Chen B., Wu Z., Tian M., Feng T., Yuanwei C., Luo X. (2020). Effect of surface morphology change of polystyrene microspheres through etching on protein corona and phagocytic uptake. J. Biomater. Sci. Polym. Ed..

[27] Bilardo R., Traldi F., Vdovchenko A., Resmini M. (2022). Influence of surface chemistry and morphology of nanoparticles on protein corona formation. WIREs Nanomed. Nanobiotechnol..

[28] Djebara M., Stoquert J.P., Abdesselam M., Muller D., Chami A.C. (2012). FTIR analysis of polyethylene terephthalate irradiated by MeV He+. Nucl. Instrum. Methods Phys. Res. Sect. B Beam Interact. Mater. At..

[29] Guan M., Jin H., Wei W., Yan M. (2023). Degradation of polyethylene terephthalate (PET) and polypropylene (PP) plastics in seawater. DeCarbon.

[30] Montazer Z., Habibi Najafi M.B., Levin D.B. (2020). Challenges with Verifying Microbial Degradation of Polyethylene. Polymers.

[31] Sandt C., Waeytens J., Deniset-Besseau A., Nielsen-Leroux C., Réjasse A. (2021). Use and misuse of FTIR spectroscopy for studying the bio-oxidation of plastics. Spectrochim. Acta Part A Mol. Biomol. Spectrosc..

[32] Maruyama T., Katoh S., Nakajima M., Nabetani H., Abbott T.P., Shono A., Satoh K. (2001). FT-IR analysis of BSA fouled on ultrafiltration and microfiltration membranes. J. Membr. Sci..

[33] Yu L., Zhang L., Sun Y. (2015). Protein behavior at surfaces: Orientation, conformational transitions and transport. J. Chromatogr. A.

[34] Mutić T., Mutić J., Ilić M., Jovanović V., Aćimović J., Andjelković B., Stanić-Vucinić D., de Guzman M.K., Andjelkovic M., Turkalj M. (2024). The Global Spread of Microplastics: Contamination in Mussels, Clams, and Crustaceans from World Markets. Foods.

[35] Samak N.A., Jia Y., Sharshar M.M., Mu T., Yang M., Peh S., Xing J. (2020). Recent advances in biocatalysts engineering for polyethylene terephthalate plastic waste green recycling. Environ. Int..

[36] Chen Z., Hay J.N., Jenkins M.J. (2012). FTIR spectroscopic analysis of poly(ethylene terephthalate) on crystallization. Eur. Polym. J..

[37] Mohamed Nor N.H., Kooi M., Diepens N.J., Koelmans A.A. (2021). Lifetime Accumulation of Microplastic in Children and Adults. Environ. Sci. Technol..

[38] Alimi O.S., Farner Budarz J., Hernandez L.M., Tufenkji N. (2018). Microplastics and Nanoplastics in Aquatic Environments: Aggregation, Deposition, and Enhanced Contaminant Transport. Environ. Sci. Technol..

[39] Prata J.C., Sequeira I.F., Monteiro S.S., Silva A.L.P., da Costa J.P., Dias-Pereira P., Fernandes A.J.S., da Costa F.M., Duarte A.C., Rocha-Santos T. (2021). Preparation of biological samples for microplastic identification by Nile Red. Sci. Total Environ..

[40] Prabhu K., Ghosh S., Sethulekshmi S., Shriwastav A. (2024). In vitro digestion of microplastics in human digestive system: Insights into particle morphological changes and chemical leaching. Sci. Total Environ..

[41] Brouwer H., Porbahaie M., Boeren S., Busch M., Bouwmeester H. (2024). The in vitro gastrointestinal digestion-associated protein corona of polystyrene nano- and microplastics increases their uptake by human THP-1-derived macrophages. Part. Fibre Toxicol..

[42] Li Z., Huang Y., Zhong Y., Liang B., Yang X., Wang Q., Sui H., Huang Z. (2023). Impact of food matrices on the characteristics and cellular toxicities of ingested nanoplastics in a simulated digestive tract. Food Chem. Toxicol..

[43] Zhang C., Zhou Z., Xi M., Ma H., Qin J., Jia H. (2025). Molecular modeling to elucidate the dynamic interaction process and aggregation mechanism between natural organic matters and nanoplastics. Eco-Environ. Health.

[44] Maurya A., Bhattacharya A., Khare S.K. (2020). Enzymatic Remediation of Polyethylene Terephthalate (PET)-Based Polymers for Effective Management of Plastic Wastes: An Overview. Front. Bioeng. Biotechnol..

[45] Świderek K., Velasco-Lozano S., Galmés M.À., Olazabal I., Sardon H., López-Gallego F., Moliner V. (2023). Mechanistic studies of a lipase unveil effect of pH on hydrolysis products of small PET modules. Nat. Commun..

