# Supplementary material for: Protein Corona Stability and Removal from PET Microplastics: Analytical and Spectroscopic Evaluation in Simulated Intestinal Conditions
Source: Foods. 2025 Oct 10;14(20):3454. doi: 10.3390/foods14203454 (PMC12562956; doi:10.3390/foods14203454)
Supplement: Supplementary file 1 [file foods-14-03454-s001.zip › foods-3855968-supplementary.pdf]

Supplementary Material

Protein Corona Stability and Removal from PET Microplastics: Analytical and Spectroscopic Evaluation in Simulated Intestinal Conditions

Tamara Lujic[1†], Tamara Mutic[1†], Ana Simovic[1], Tamara Vasovic[1], Stefan Ivanovic[2], Maja Krstic Ristivojevic[1], Vesna Jovanovic[1], Tanja Cirkovic Velickovic[1,3*]

[1]Center of Research Excellence in Molecular Food Sciences and Department of Biochemistry, University of Belgrade — Faculty of Chemistry, Studentski trg 12-16, 11000 Belgrade, Serbia

[2]Institute of Chemistry, Technology and Metallurgy, National Institute of the Republic of Serbia, Department of Chemistry, University of Belgrade, Njegoseva 12, 11000, Belgrade, Serbia;

[3]Serbian Academy of Sciences and Arts, Knez Mihailova 35, 11000 Belgrade, Serbia

[†]These authors contributed equally to this work

[*] tcirkov@chem.bg.ac.rs

S1. Microscopy based assesment of hard corona of PET MPs after Nile red staining

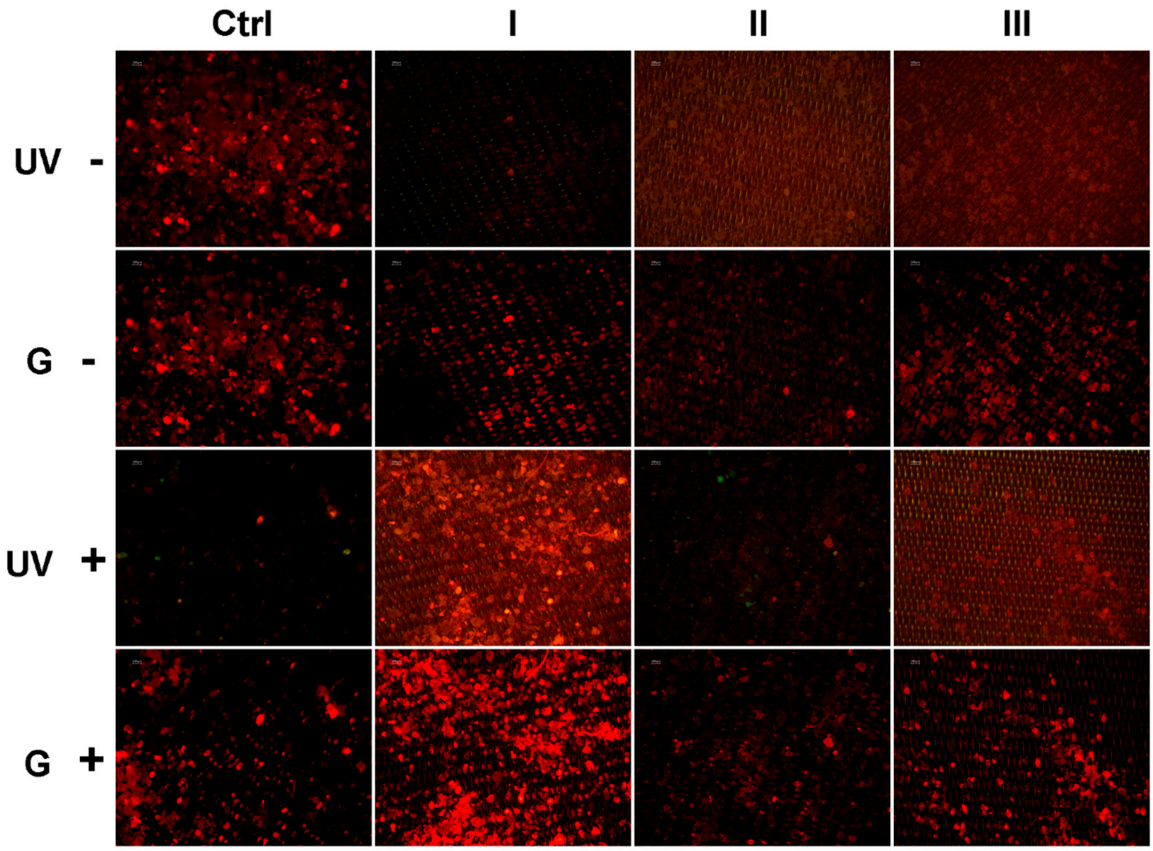

Figure S1. Fluorescence images of PET MPs with (Ctrl+) or without (Ctrl-) BSA-AF488 hard corona after staining with Nile red following treatment with three clean-up protocols: I (10% SDS + 15% H2O2); II (2 x 30% H2O2); III (15% H2O2 + 10% KOH). Ctrl+ and Ctrl-: Positive and negative control (PET MPs with or without BSA-AF488 hard corona without any treatments. Images were acquired with 4× magnification after excitation at 340–380 nm (UV filter for BSA-AF488) and/or 527.5–552.5 nm (G – green filter for Nile Red).

Section S2. Residual protein presence evaluation in control samples of PET MPs

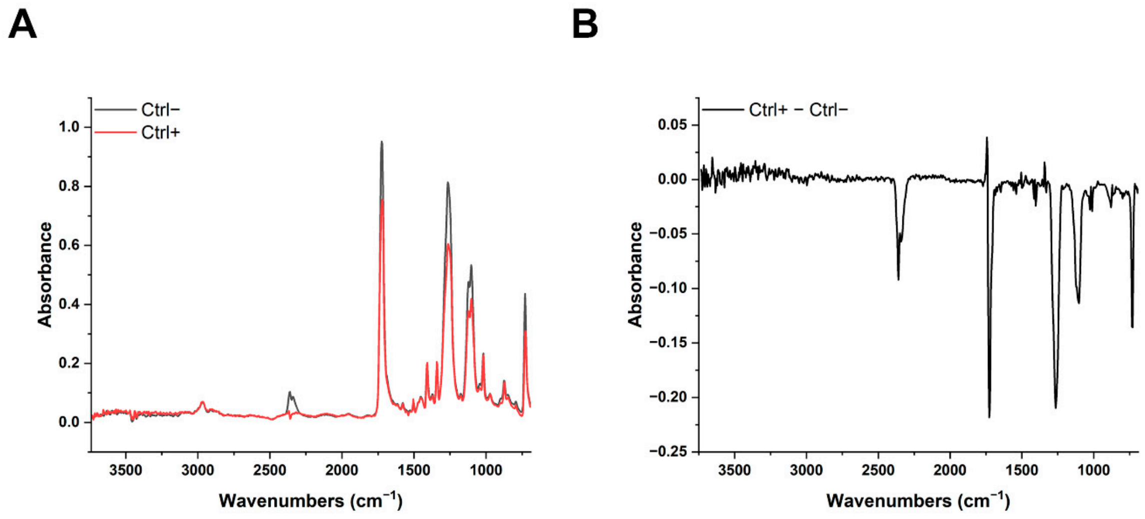

Figure S2. Overlaid spectra (A) and comparison of difference spectra (B) of PET MPs with BSA-AF488 hard corona (Ctrl+) and untreated PET MPs (Ctrl-).

S3. PET MPs exposure to digestive enzymes

S3.1. ATR-FTIR analysis of PET MPs

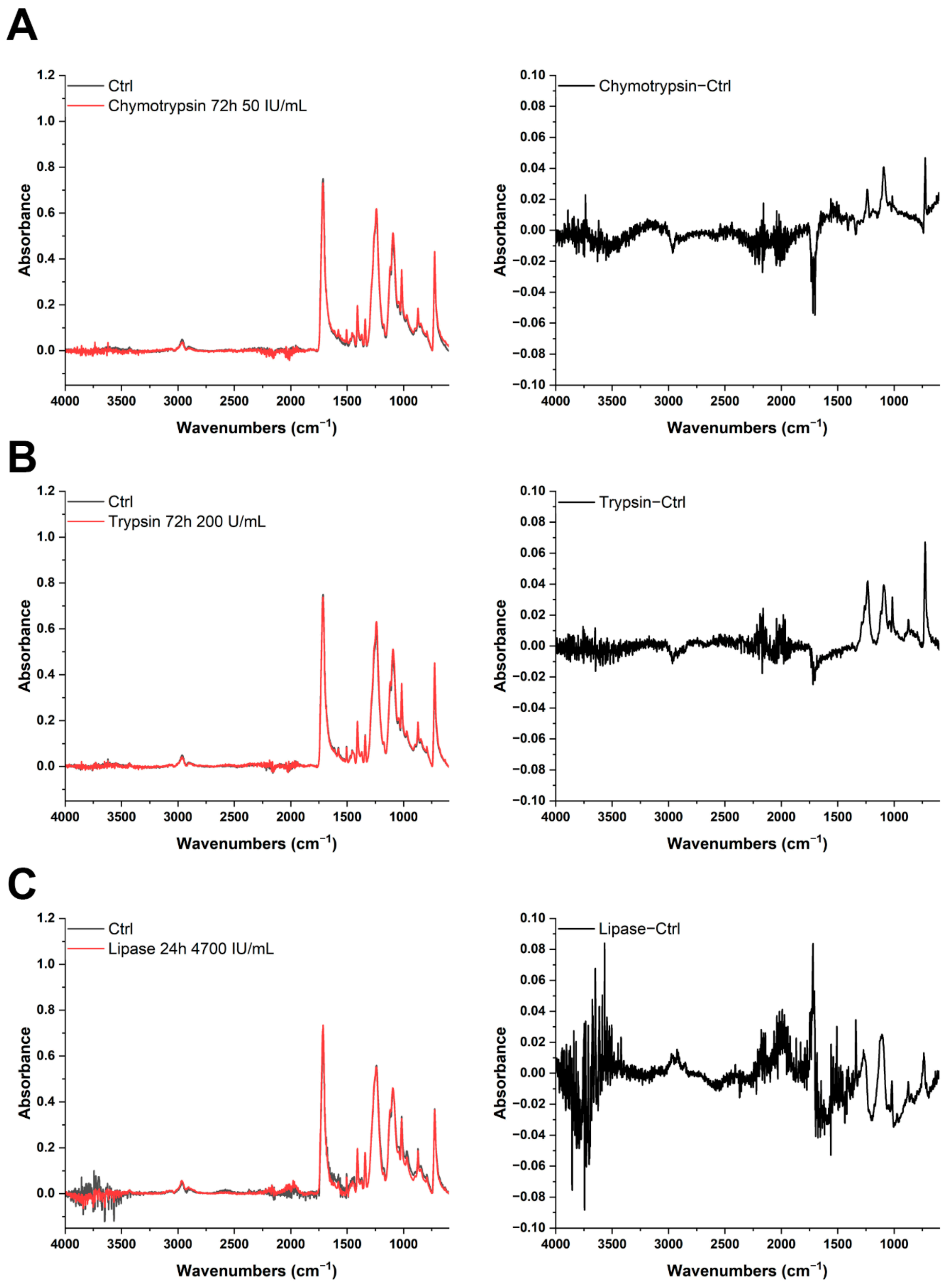

Figure S3. Overlaid spectra (left) and the difference spectra (right) of PET MPs incubated: 72h with chymotrypsin 50 IU/ml (A), 72h with trypsin 200 IU/mL (B) and 24h with lipase 4700 IU/mL (C) in simulated intestinal fluid and corresponding controls (PET MPs incubated without enzymes under the same conditions). Clean-up protocols based on SDS + H_2O_2 for chymotrypsin and trypsin and protocol based on H_2O_2 + KOH were used for removing hard and soft coronas.

S3.2. SDS-PAGE analysis of digestive enzymes

All enzyme preparations were tested for their purity and protein content on a 14% polyacrylamide gel. In order to compare them, the same amount of preparations was applied in each lane.

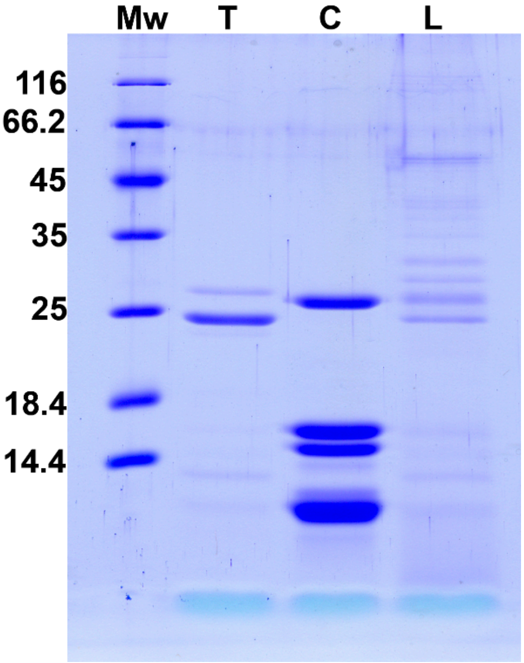

Figure S4. SDS-PAGE of digestive enzymes used in the experiments on 14% polyacrylamide gel. Mw – molecular weight marker; T – trypsin; C – chymotrypsin; L-lipase.

S3.3. Testing for presence of microbiological contamination

Samples incubated for prolonged periods with digestive enzymes were tested for the presence of microbiological contamination. Briefly, 100 µL of each sample was plated on nutrient agar. Microorganism were left to grow for 24 h at 37°C and visually observed.

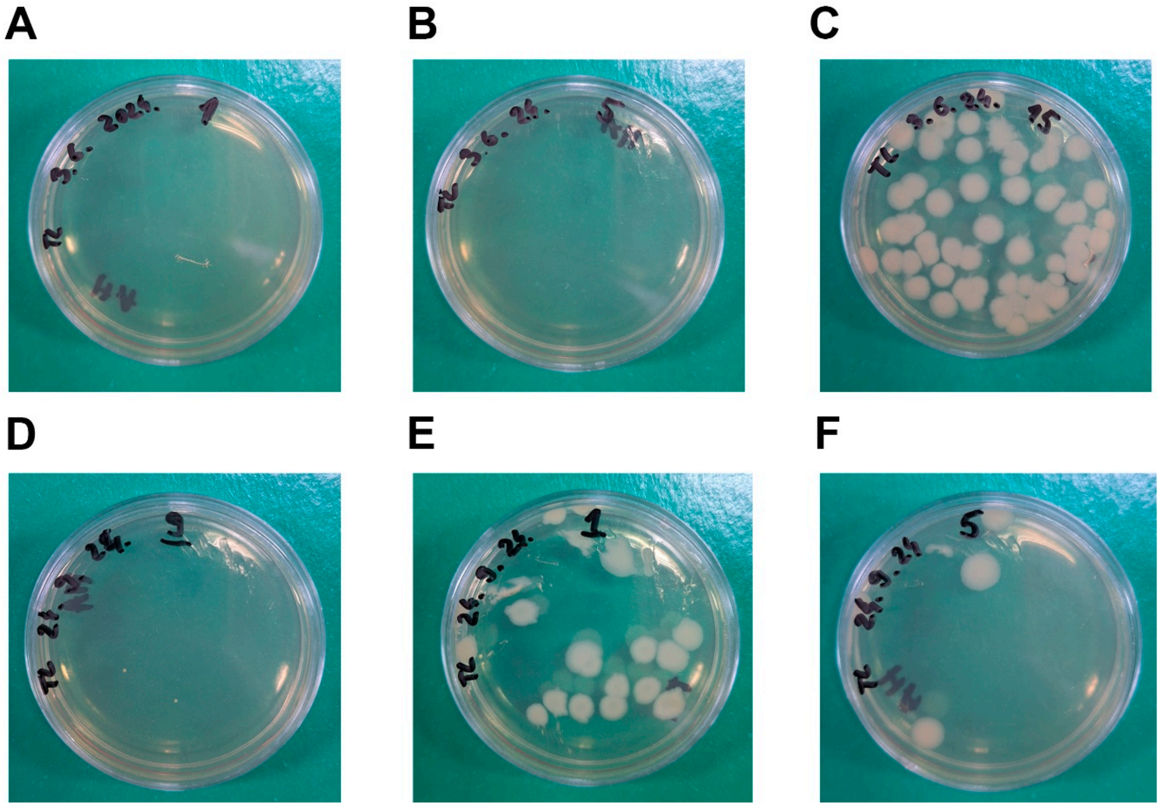

Figure S5. Testing of microbial growth for PET MP samples incubated with intestinal enzymes. A – SIF after 24 h; B – 25 U/mL chymotrypsin after 24 h; C – 930 U/mL lipase after 24 h; D – SIF after 72 h; E – 50 U/mL chymotrypsin after 72 h; F – 200 U/mL trypsin after 72h.
